# A novel patient-derived orthotopic xenograft (PDOX) mouse model of highly-aggressive liver metastasis for identification of candidate effective drug-combinations

**DOI:** 10.1038/s41598-020-76708-9

**Published:** 2020-11-18

**Authors:** Zhiying Zhang, Kaiwen Hu, Kentaro Miyake, Tasuku Kiyuna, Hiromichi Oshiro, Sintawat Wangsiricharoen, Kei Kawaguchi, Takashi Higuchi, Sahar Razmjooei, Masuyo Miyake, Sant P. Chawla, Shree Ram Singh, Robert M. Hoffman

**Affiliations:** 1 AntiCancer, Inc., San Diego, CA USA; 2 Department of Surgery, University of California, San Diego, CA USA; 3 Department of Oncology, Dongfang Hospital, Beijing University of Chinese Medicine, Beijing, China; 4 Sarcoma Oncology Center, Santa Monica, CA USA; 5 Basic Research Laboratory, National Cancer Institute, Frederick, MD USA

**Keywords:** Chemotherapy, Cancer therapy, Gastrointestinal cancer, Cancer, Cancer models

## Abstract

Liver metastasis is a recalcitrant disease that usually leads to death of the patient. The present study established a unique patient-derived orthotopic xenograft (PDOX) nude mouse model of a highly aggressive liver metastasis of colon cancer. The aim of the present study was to demonstrate proof-of-concept that candidate drug combinations could significantly inhibit growth and re-metastasis of this recalcitrant tumor. The patient’s liver metastasis was initially established subcutaneously in nude mice and the subcutaneous tumor tissue was then orthotopically implanted in the liver of nude mice to establish a PDOX model. Two studies were performed to test different drugs or drug combination, indicating that 5-fluorouracil (5-FU) + irinotecan (IRI) + bevacizumab (BEV) and regorafenib (REG) + selumetinib (SEL) had significantly inhibited liver metastasis growth (*p* = 0.013 and *p* = 0.035, respectively), and prevented liver satellite metastasis. This study is proof of concept that a PDOX model of highly aggressive colon-cancer metastasis can identify effective drug combinations and that the model has future clinical potential.

## Introduction

Liver metastasis is a major cause of death for common cancers and is very frequent in colon cancer^1^. Surgery for liver metastasis does not usually lead to cures^1^. For colon cancer, chemotherapy including FOLFIRI, the combination of folinic acid (FOL), fluorouracil (5-FU) and irinotecan (IRI); FOLFOX, the combination of FOL, 5-FU and oxaliplatum (OXA); CAPEOX, the combination of capecitabine (CAPE) and OXA; or FOLFOXIRI, the combination of FOL, 5-FU, OXA and IRI plus targeted therapy such as bevacizumab (BEV), regorafenib (REG), panitumumab (PAN) or cetuximab (CET) are used to treat liver metastasis^1–3^. The outcome of such chemotherapy is variable and appears to be patient specific^3–6^. Yondelis (trabectedin, YON) is an alkylating drug for the treatment of patients with unresectable or metastatic liposarcoma or leiomyosarcoma who received a prior anthracycline-containing regimen^7^. Selumetinib (SEL) is a non-ATP-competitive mitogen-activated protein kinase 1 and 2 (MEK1 and MEK2) inhibitor^8^. YON and SEL have not yet been used for metastatic colon cancer in the clinic.

In order to develop individualized precision treatment for cancer, we have developed the patient-derived orthotopic xenograft (PDOX) mouse model. Tumor fragments from the patient are implanted into the corresponding anatomic location (orthotopic) in the nude mouse via surgical orthotopic implantation (SOI). We have successfully established PDOX models of all major cancer types^9–22^. We demonstrated that the PDOX model is superior to the subcutaneous patient-derived xenograft (PDX) model due to the proper tumor microenvironment which enables tumor invasion and metastasis^23,24^, matching the patient metastatic pattern^14,25^.

In the present study, we established a PDOX model of a highly-aggressive colon cancer liver metastasis by implanting a fragment of a highly metastatic colon cancer directly into the liver in the nude mouse. The PDOX model enabled the identification of two candidate effective combination regimens that arrested liver metastasis growth and prevented satellite-metastasis formation, and demonstrated proof-of-concept that such combinations could be identified with this unique PDOX model of highly-metastatic patient colon cancer.

## Materials and methods

### Mice

Athymic nude mice (AntiCancer, Inc., San Diego, CA), 4–6 weeks old, were used^17–23^. Procedures for mouse housing, handling, anesthesia, feeding, and humane endpoint criteria have been previously described^17–23^. In order to minimize any suffering of the animals, anesthesia and analgesics were used for all surgical experiments as previously described^17–23^. The animal studies were conducted in compliance with an AntiCancer, Inc. Institutional Animal Care and Use Committee (IACUC) protocol exclusively approved for this study and in accordance with the principles and procedures outlined in the National Institutes of Health Guide for the Care and Use of Animals under Assurance Number A3873-1^21^.

### Patient-derived tumor

A patient diagnosed with colon cancer liver metastasis had the metastatic tumor resected in Scripps Clinic, San Diego. Written informed consent was provided by the patient, and the Institutional Review Board (IRB) of Scripps Clinic approved this experiment. Experiments in the present study were performed per the Declaration of Helsinki guidelines and in agreement with national regulations for the experimental use of human material.

### Establishment of PDOX models of colon cancer liver metastasis

**Figure 1:**
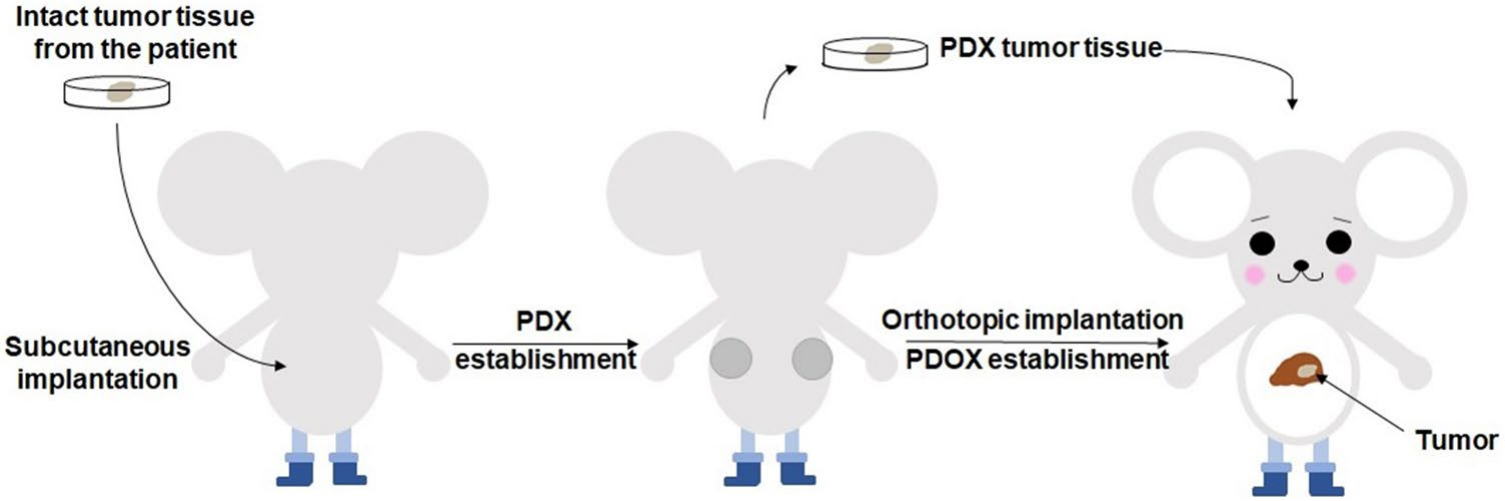
Schematic diagram of surgical orthotopic implantation (SOI) for the colon cancer liver-metastasis model. After the patient’s liver-metastasis surgery, intact liver metastasis tumor tissue from the patient was initially implanted subcutaneously into nude mice to establish a stable patient-derived xenograft (PDX) model, defined as P0. After the P0 tumor grew to approximately 1 cm in diameter, the mice were sacrificed and the subcutaneous tumors were harvested, viable tumor tissue was minced into 2 mm^3^ fragments for orthotopic transplantation into the liver of the nude mice to establish a patient-derived orthotopic xenograft (PDOX) model. Please see materials and methods for details.

After surgical resection of the patient, a fresh tumor sample was transferred immediately to the laboratory in AntiCancer, Inc. in a sterile tube with RPMI-1640 medium. The tube was put in ice to maintain the temperature at 4 °C^22^. The sample was divided into small fragments and implanted subcutaneously into both flanks of nude mice to establish a patient-derived xenograft (PDX) model. When the subcutaneous tumors grew over 10 mm in diameter, the tumors were harvested and inspected, and any suspected or grossly necrotic tissue was removed. Healthy tumor tissues were subsequently cut into small fragments of approximately 2 mm^3^. After anesthesia, a 1 cm skin incision was made along with the anterior abdominal midline of the nude mouse using surgical scissors. The liver lobe was exposed, the capsule at the transplantation site was stripped, and one tumor fragment was implanted and fixed with 8–0 surgical sutures (nylon). The liver was replaced into the abdomen and the abdomen was closed with 6–0 surgical sutures (Ethilon, Ethicon, Inc., NJ) (Fig. 1)^22^.

### Treatment study design in the colon-cancer liver-metastasis PDOX model

The tumor volume and body weight were measured every 2 weeks after laparotomy using electronic calipers and an electronic scale, respectively. Calipers could be used since the tumor was growing mostly on the surface of the liver. The tumor volume was estimated by measuring the perpendicular minor dimension (W) and major dimension (L). Approximate tumor volume was calculated by the formula (W^2^ × L)/2. When the volume of tumors reached approximately 60  mm^3^, the mice were randomized into 4 groups. For the first study, the mice with liver-implanted tumors were divided as follows: Group1, untreated control (n = 5); Group 2, 5-FU + IRI + BEV (n = 6); Group 3, 5-FU + OXA + BEV (n = 6); and Group 4, 5-FU + BEV (n = 5). The treatment period was 4 weeks. For the second study, when the volume of tumors reached approximately 60 mm^3^, the mice with liver-implanted tumors were also randomized into 4 groups: Group1, untreated control (n = 6); Group 2, YON + OXA (n = 4); Group 3, REG alone (n = 7); and Group 4, SEL + REG (n = 5). There were 6 mice in each group. The treatment period was 2 weeks. The treatment protocol is shown in Table 1. The drug doses were determined by published literature^26–38^. At the end of the study, the mice were euthanized by CO_2_ inhalation, the tumors were harvested, and directly measured. The images of the mice and the tumors were obtained with the OV100 Small Animal Imaging System (Olympus Corporation, Tokyo, Japan)^22^.

**Table 1:** Treatment protocol for the colon-cancer liver-metastasis PDOX model. The mice were randomized into 4 different groups. For study one, the treatment period was 4 weeks. For study two, the treatment period was 2 weeks.

Group	Treatment	Route	Dose	Frequency
**Study 1**				
1	Control	ip	200 μL (PBS)	Once a week
2	5-Fluorouracil	ip	50 mg/kg	Once a week
	Irinotecan	ip	40 mg/kg	Twice a week
	Bevacizumab	ip	5 mg/kg	Twice a week
3	5-Fluorouracil	ip	50 mg/kg	Once a week
	Oxaliplatum	ip	6 mg/kg	Once a week
	Bevacizumab	ip	5 mg/kg	Twice a week
4	Fluorouracil	ip	50 mg/kg	once a week
	Bevacizumab	ip	5 mg/kg	Twice a week
**Study 2**				
1	Control	ip	200 μL (PBS)	Once a week
2	Yondelis	iv	0.15 mg/kg	Once a week
	Oxaliplatum	ip	6 mg/kg	Twice a week
3	Regorafenib	po	30 mg/kg	Once a day
4	Selumetinib	po	20 mg/kg	Once a day
	Regorafenib	po	30 mg/kg	Once a day

### Histological examination

Freshly-harvested tumors and liver tissue were fixed in 10% formalin and embedded in paraffin for further histology analysis. Tissue sections (5 mm) were deparaffinized in ClearRite and rehydrated in an ethanol series. Hematoxylin and eosin (H and E) staining was performed according to standard protocols^22^. Histological examination was performed with a BHS System Microscope (Olympus Corporation)^22^. Histological images were obtained with INFINITY ANALYZE 7 software (Lumenera Corporation, Ottawa, Canada)^22^, no software license is needed.

### Statistical analysis

IBM SPSS Statistics Version 24.0 (IBM, New York City, NY) was used for statistical analyses in this study. The Shapiro–Wilk test was used to assess the normal distribution. Bartlett’s test was used to verify the homogeneity of variances among groups. One-way ANOVA with Tukey HSD for post hoc analysis was used for the parametric test, and the Kruskal–Wallis one-way ANOVA with Steel–Dwass for post hoc analysis was used for the non-parametric comparison. Data are shown as mean ± standard deviation (SD). A *p* value of 0.05 or less indicates statistical significance.

## Results

### Orthotopic tumor growth in the liver

**Figure 2:**
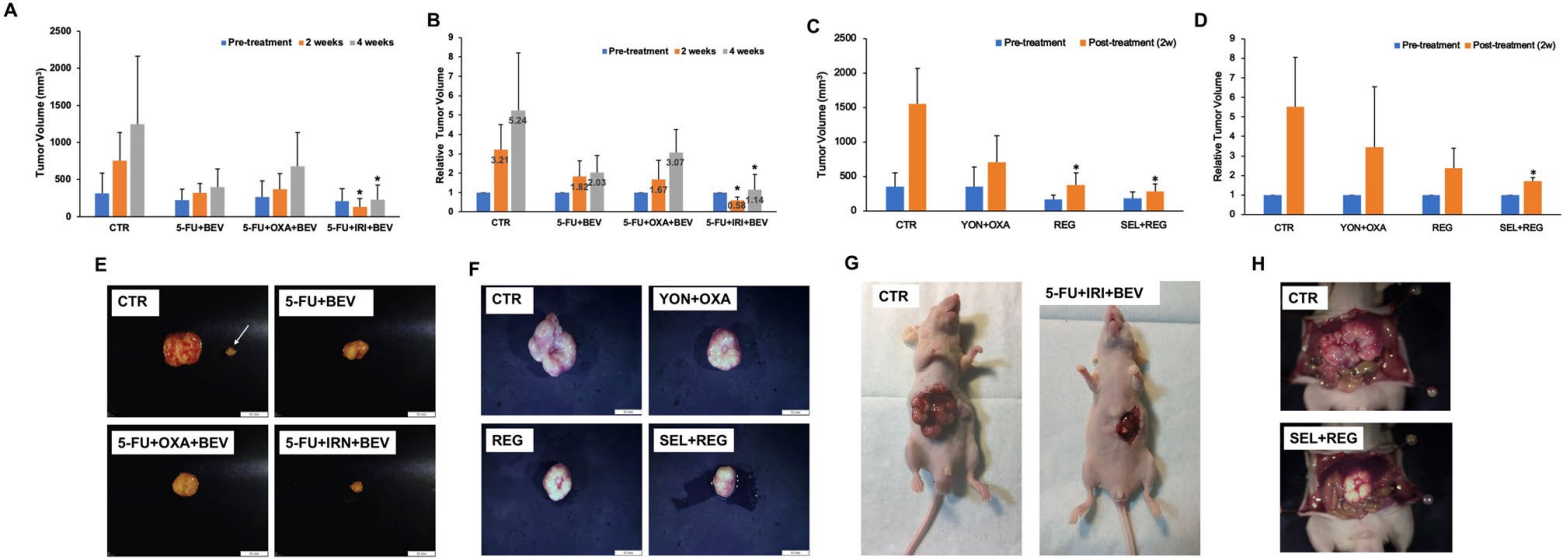
Drug efficacy testing. For the first study, untreated control (n = 5); 5-FU + BEV (n = 6), 5-FU + OXA + BEV (n = 6); 5-FU + IRI + BEV (n = 5). For the second study, untreated control (n = 6); YON + OXA (n = 4), REG (n = 7); SEL + REG (n = 5). (**A**) Study 1, comparison of tumor volume before treatment; at 2 weeks; and after treatment. (**B**) Study 1, comparison of relative tumor volume before treatment; at 2 weeks; and after 4 weeks. (**C)** Study 2, comparison of tumor volume before treatment and after treatment. (**D**) Study 2, comparison of relative tumor volume before treatment and after treatment. The data are shown as mean + standard deviation (SD). (**E**) Study 1, representative images of a harvested tumor in each group. The white arrow shows the intrahepatic metastasis along with the primary implanted tumor. Scale bar: 10 mm. (**F**) Study 2, representative images of harvested tumor in each group. (**G**): Study 1, representative images of the control and treated tumors. (**H**) Study 2, representative images of the control and treated tumors.

The untreated PDOX tumor grew extremely aggressively in the control-group mice, mimicking the liver metastasis in the patient (Fig. 2G,H).

### Drug efficacy in the colon-cancer liver-metastasis PDOX model

In the first study, the two-week measurement made in situ with calipers is consistent with the endpoint measurement of the harvested tumor at 4 weeks. The relative tumor volume in the various groups, which is the ratio of the post-treatment tumor volume to pre-treatment tumor volume, was as follows after the 4-week treatment period: Group 1, control: 5.24 ± 2.97; Group 2, 5-FU + BEV: 2.03 ± 0.88; Group 3, 5-FU + OXA + BEV: 3.07 ± 1.19; Group 4, 5-FU + IRI + BEV: 1.13 ± 0.80. Only Group 4, 5-FU + IRI + BEV significantly inhibited the growth of the colon cancer liver metastasis in the PDOX model compared with the untreated control (*p* = 0.013). The growth of the liver metastasis was arrested by the combination of 5-FU + IRI + BEV (Fig. 2A,B).

In the second study, the relative tumor volume was as follows after the 2-week treatment period: Group 1, control: 5.53 ± 2.52; Group 2, YON + OXA: 3.46 ± 3.09; Group 3, REG: 2.38 ± 1.01; Group 4, SEL + REG: 1.7 ± 0.19. Only Group 4, SEL + REG significantly inhibited the growth of the colon cancer liver metastasis in the PDOX model compared with the untreated control (*p* = 0.035). The growth of the liver metastasis was almost arrested by the combination of SEL + REG (Fig. 2C,D).

### Drug efficacy on satellite metastasis

In the first study, intrahepatic metastases were observed in Group 1, control (3 mice, 10 metastases); Group 2, 5-FU + BEV (1 mouse, 1 metastasis) and Group 3, 5-FU + OXA + BEV (2 mice, 2 metastases). Group 4, 5-FU + IRI + BEV prevented intra-hepatic metastases, as well as arrested tumor growth. (Table 2, Fig. 2E,G).

**Table 2:** Intra-hepatic satellite metastases. Total satellite metastases were identified at necropsy.

Group	Mouse number with metastasis	Total number of satellite metastasis in the group
**Study 1**		
Control	3	10
5-FU + BEV	1	1
5-FU + OXA + BEV	2	2
5-FU + IRI + BEV	0	0
**Study 2**		
Control	1	6
YON + OXA	1	2
REG	2	3
SEL + REG	0	0

In the second study, intrahepatic metastases were observed in Group 1, control (1 mouse, 6 metastases); Group 2, YON + OXA (1 mouse, 2 metastases) and Group 3, REG (2 mice, 3 metastases) (Table 2). Group 4, SEL + REG prevented intra-hepatic metastases, as well as almost arresting tumor growth. (Table 2, Fig. 2F,H).

### Effect of drugs on mouse body weight

**Figure 3:**
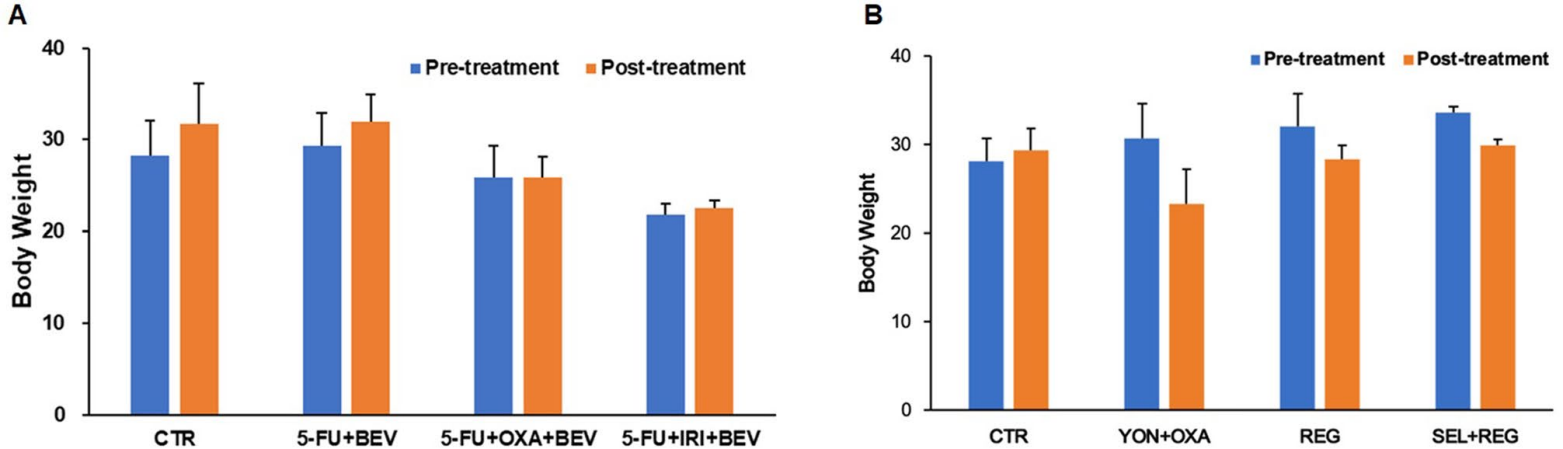
Effect of drugs on body weight. The Kruskal–Wallis one-way ANOVA test was used to analyze the differences between each group. The data are shown as mean + SD.

We measured the mouse body weight every two weeks to monitor the overt toxicity of the drugs. There was no significant body-weight loss in any group at the end of this study. (Fig. 3A,B).

### Effect of drugs on tumor histology

**Figure 4:**
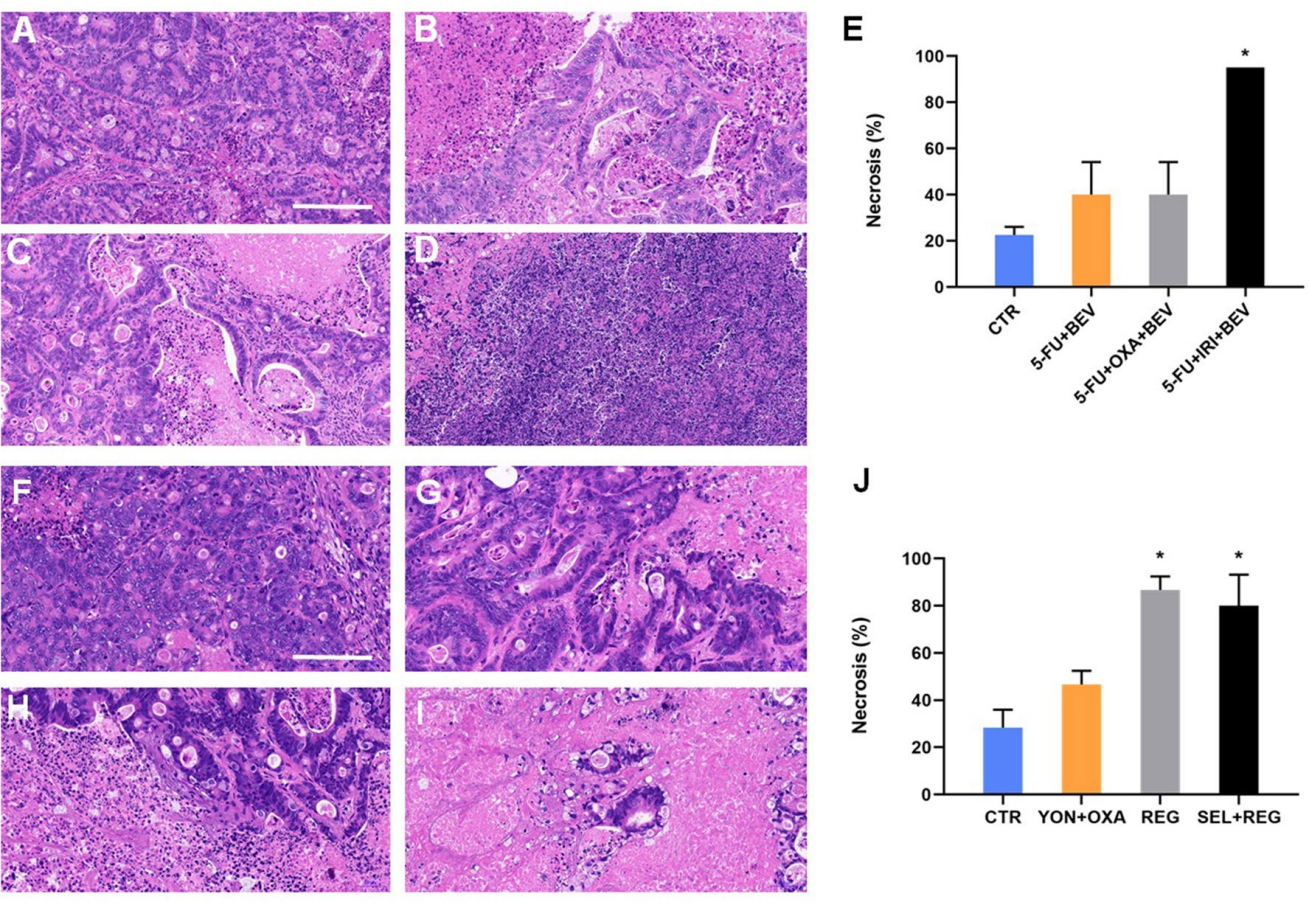
Effect of drugs on tumor histology. (**A**) Control group. (**B**) 5-FU + BEV group. (**C**): 5-FU + OXA + BEV group. (**D**): 5-FU + IRI + BEV group. (**E**) Extent of tumor necrosis. (**F**) Control group. (**G**) YON + OXA group. (**H**): REG group. (**I**) SEL + REG group. (**J**) Extent of tumor necrosis. *Mean *p* < 0.05. Scale bar: 200 μm. Images were obtained with INFINITY ANALYZE 7 software, https://www.lumenera.com/products/microscopy/infinity-analyze.html.

We analyzed the tumor histology in each group to examine whether the drug combinations could induce necrosis (Fig. 4). In the first study, Group 1, control, showed a classic morphology for colon adenocarcinoma with pleomorphism and a small extent of necrosis. Group 2, 5-FU + BEV, showed a moderate extent of treatment-related tumor necrosis. There was a similar degree of treatment-related tumor necrosis in Group 3, 5-FU + OXA + BEV. In contrast, in Group 4, 5-FU + IRI + BEV, the tumor had extensive treatment-related necrosis. In the first study, treatment efficacy, measured by tumor necrosis, was significantly higher in the 5-FU + IRI + BEV group than the 5-FU + OXA + BEV; 5-FU + BEV; and control groups (*p* < 0.05) (Fig. 4E). In the second study, Group 1, control, also showed a classic morphology for colon adenocarcinoma with pleomorphism and a small extent of necrosis. Group 2, YON + OXA, showed a moderate extent of treatment-related tumor necrosis. In Group 3, REG and Group 4, SEL + REG, the tumor had extensive treatment-related necrosis. Treatment efficacy measured by tumor necrosis was significantly higher in the REG alone group and SEL + REG group than the YON + OXA and control groups (*p* < 0.05). (Fig. 4J).

## Discussion

In the present report, we found that the combination of 5-FU, IRI and BEV as well as the combination of SEL and REG showed the best efficacy on the liver-metastasis PDOX, arresting or almost arresting liver-metastasis growth and preventing satellite metastases within the liver. The combination of 5-FU, OXA and BEV, the combination of 5-FU and BEV, the combination of YON and OXA as well as REG alone did not show significant anti-tumor efficacy. The combination of 5-FU, IRI and BEV, and the combination of SEL and REG and REG alone demonstrated a high level of tumor necrosis (*p* < 0.05), indicating a high level of cancer-cell killing. This PDOX model for this liver-metastatic colon cancer thus clearly digstinguished two candidate effective combinations for this highly-aggressive liver-metastasis PDOX.

Intrahepatic satellite metastases occurred in this study, which also demonstrated the power of the PDOX model, in this case to identify the extreme malignancy of the original tumor (Fig. 2G,H), and then target it with appropriate therapy. Subcutaneous PDX models very rarely develop metastasis^24,25^. Since this is a liver metastasis of colon cancer, the microenvironment in the liver enabled the aggressiveness of the tumor to be expressed^39,40^.

The effective drug combinations were identified 7 months and 12 months after the surgery, in study 1 and 2 respectively, demonstrating the potential of PDOX model for the clinician to decide optimal second-line therapy, which is often needed as first-line therapy for colon-cancer metastasis often fails. Future studies will correlate drug response in the PDOX model and the patient.

## Conclusion

The present report was on a PDOX model of an extremely aggressive liver-metastasis model, that could grow extensively in the liver to form a large tumor as well as form satellite re-metastasis in the liver. The present study showed it was possible to inhibit this aggressive metastasis tumor with combination chemotherapy, which was proof-of-concept that this model could be used to identify new combination therapy for liver metastasis as well as for 2nd-line or later therapy for patients in the future.
